# How evaluative pairings improve body dissatisfaction in adult women: evidence from a randomized-controlled online study

**DOI:** 10.1186/s40337-024-00975-4

**Published:** 2024-01-24

**Authors:** Katharina Dumstorf, Georg Halbeisen, Georgios Paslakis

**Affiliations:** University Clinic for Psychosomatic Medicine and Psychotherapy, Medical Faculty, Campus East-Westphalia, Ruhr-University Bochum, Virchowstr. 65, 32312 Luebbecke, Germany

**Keywords:** Evaluative conditioning, Body image, Eating disorders, Contingency learning, Psychotherapy, Pairing procedures

## Abstract

**Background:**

Many young women are dissatisfied with their bodies. This study investigated the effect on current body dissatisfaction levels of a newly developed evaluative conditioning procedure that paired self-similar and self-dissimilar images of bodies with positive and neutral affective images, respectively. We hypothesized that learning the contingency that self-similar bodies predict positive affectivity is one process that could aid in explaining how these procedures function.

**Methods:**

Adult women without disordered eating pathology participated in an online experiment with random assignment to an intervention or a control condition. All participants initially rated body images in self-similarity and were subsequently asked to categorize positive and neutral images by valence as quickly and accurately as possible. In the intervention condition, self-similar bodies systematically preceded positive images, and self-dissimilar images preceded neutral images, creating a *similar body → positive* contingency. Pairings in the control condition were unsystematic such that no contingency was present. We measured categorization latencies and accuracies to infer contingency learning as well as current body dissatisfaction immediately before and after exposure to the pairings. All participants further completed measures of trait body image concerns and disordered eating psychopathology at baseline, which we examined as moderators of an expected relation between condition assignment, contingency learning, and body dissatisfaction improvements.

**Results:**

We analyzed data from N = 173 women fulfilling the inclusion criteria. Moderated mediation analyses showed that assignment to the intervention (vs. control) condition predicted increased *similar body → positive* contingency learning, which in turn predicted improved body dissatisfaction post-intervention, but only among women with higher pre-existing trait body image concerns or disordered eating levels.

**Conclusions:**

The findings point toward the relevancy of further exploring the utility of pairing procedures. *Similar body → positive* contingency learning predicted improved body dissatisfaction in individuals with normatively high body image concerns, which suggests pairing procedures could help inform future research on reducing body dissatisfaction.

**Supplementary Information:**

The online version contains supplementary material available at 10.1186/s40337-024-00975-4.

## Abstract (plain language summary)

Many people are dissatisfied with how their bodies look or how much they weigh. Body dissatisfaction can increase the risk of developing an eating disorder. This study tested a method for reducing body dissatisfaction among women. The method included pairing pictures of bodies judged as similar to one’s own body with positive pictures. For one half of the study participants, we arranged the pairings in a way that one could systematically learn that similar body pictures and positive pictures go together. Compared to the other half of study participants who were shown pairings by chance, we found that study participants indeed learned that similar body pictures and positive pictures go together. Moreover, this learning made participants who were generally dissatisfied with their bodies or who reported disordered eating symptoms more satisfied with their bodies immediately after the procedure. These findings suggest that the method could be further developed, investigated, and used in treating or preventing eating disorders.

**Supplementary Information:**

The online version contains supplementary material available at 10.1186/s40337-024-00975-4.

## Introduction

Many young women are dissatisfied with their bodies, including body shape, size, muscularity, and weight [1, 2]. Body dissatisfaction can originate from internalizing unrealistic body ideals [3], comparisons on social media [4], and has been associated with childhood maltreatment [5]. Substantial levels of body dissatisfaction affect between 11 and 72% of women [6, 7], and pose an increased risk for disordered eating and weight-control behaviors [8, 9], lower rates of health-promoting behaviors (e.g., cancer screenings [10], and even increased mortality [11–13]. Given these potentially severe consequences, investigating novel techniques to alleviate body dissatisfaction is crucial.

Existing standalone interventions for improving body dissatisfaction frequently target antecedent and maintaining factors of negative body images. These can be grouped into cognitive-behavioral techniques (e.g., cognitive restructuring, exposure exercises), interventions providing media literacy (e.g., discussing beauty ideals), enhancing general self-esteem (e.g., social comparison exercises), and psychoeducation [14]. Although overall effective [14], the effect sizes corrected for publication bias of body dissatisfaction improvements found in a comprehensive meta-analysis were small (i.e., Cohen’s *d* = 0.15, 95% CI [0.02; 0.28]), indicating a need to develop and explore further interventions.

A promising candidate for improving body dissatisfaction directly is evaluative conditioning or *pairing procedures* [15]. Evaluative conditioning has been defined as the change in liking (i.e., the subjective appraisal as positive or negative) of a target conditioned stimulus (CS) due to its pairing with a liked or disliked source, i.e., the unconditioned stimulus (US; [16]. In a prototypical study, target stimuli such as artificial brand names are repeatedly presented for a few seconds with either positive or negative source stimuli (e.g., pictures, words). Statistically, the repeated pairings create a contingency between two events (the occurrence of a target stimulus predicts the occurrence of a positive or negative source stimulus). The pairings typically lead to changes in the evaluation of the targets, usually assessed immediately afterwards, such that targets paired with liked sources are evaluated more favorably compared to both unpaired targets and targets paired with disliked sources [17].

Given that pairing procedures have been frequently used to change liking across different domains [18, 19], Martijn et al. [20] adapted a pairing procedure to improve current (state-like) body dissatisfaction in young women. In a first session, participants’ full body photos were taken in standardized clothing and body dissatisfaction was assessed. After eight days, the photos were repeatedly paired in one condition as target stimuli (CS) with smiling faces as evaluative sources (US; other body images were paired with neutral and frowning faces as control target and source stimuli, respectively). The control condition consisted of unsystematic pairings in which neither the own nor other body images predicted the presentation of smiling faces. Compared to that, the *own body → positive* contingency in the intervention condition improved current body dissatisfaction levels, assessed directly after the conditioning procedure, in the small-to-medium range. However, medium-to-large increases were observed for women with high levels of pre-existing (trait-like) body image concerns. Martijn et al. [20] suggested that social recognition and acceptance could be more important for women with high, trait-like body image concerns than those with low body image concerns. Pairing one’s body with positive stimuli could effectively simulate social recognition, explaining the procedure’s pronounced effect among women with higher body image concerns.

Aspen et al. [21] and Kollei et al. [22] provide further evidence that pairing own body images with positive stimuli improves body dissatisfaction. Both studies employed pairing sessions repeatedly (over the course of four or two weeks, respectively), and demonstrated effects with prolonged follow-ups (four and twelve, or four weeks, respectively). However, several more recent findings challenge these effects. For example, pairing own body photos with smiling faces in conceptual (six pairing sessions over three weeks) and direct replication studies of the Martijn et al. procedure did not improve current body dissatisfaction among women with [23] and without eating disorders [24], irrespective of a delayed or immediate body dissatisfaction assessment. Similar studies among female university students in which own face and body pictures were paired with images of other women’s smiling faces in repeated sessions over one week [25] or emojis in a single session [26] could also not find that current body dissatisfaction improved in neither a delayed nor an immediate assessment, irrespective of the pre-existing levels of body image concerns.

While the overall heterogeneous findings could question the utility of pairing procedures for improving body dissatisfaction, it is important to note that previous studies rarely examined the mediating processes (for an overview of processes, see [17]. For example, previous studies rarely examined the role of encoding the manipulated *body → positive* contingency (i.e., contingency learning), which could be one relevant mechanism to improving body dissatisfaction [27] (notably, both Martijn et al. [20], and Glashouwer et al. [24], had participants estimate the manipulated contingency, but did not conduct mediation analyses). Indeed, selectively attending to both target and source stimuli is a pre-requisite for contingency learning [28], and several previous experimental studies show that the success of encoding the target-source contingency can predict the effectiveness of pairing procedures (e.g., [29–31]. We would therefore expect that evaluative pairing effects for improving body satisfaction could depend on learning (or failing to learn) the intended *target → source* contingency.

For example, participants in Kosinski et al. [25], who used an “app” to pair face and body pictures with images of other women’s smiling faces, may have failed to learn a *body → positive* contingency, given they were tasked with selectively attending to the identity (rather than positive valence) of a paired smiling face that was presented among other smiling faces [32]. Similarly, Glashouwer et al. [24] and Masselman et al. [26] had participants wear colored clothing that distinguished their body photos from control target stimuli. While aiding in standardizing the procedures and recognizing one’s body, this color-coding may have had the unintended side-effect of distracting attention away from relevant body features such as size or shape. Participants thus may have encoded a *color → positive* contingency instead. Although speculative and a posteriori, this line of reasoning illustrates that ensuring and assessing the encoding of a *body → positive* contingency could be crucial for a pairing procedure’s success in improving body dissatisfaction.

The present experiment investigated the effect of a newly designed pairing procedure to improve body dissatisfaction in women. Because previous studies show considerable variations in terms of “dosing” (the number of pairings and sessions) and follow-up delay (from immediate to twelve weeks after), we focused on a likely common denominator, i.e., the ability of a single pairing session to improve body dissatisfaction immediately. We thus operationalized body dissatisfaction in terms of a changeable state. Based on the idea that pairing effects could be driven by successfully encoding a *body → positive* contingency, our paradigm adopted an active evaluative pairing procedure with simultaneous contingency learning assessment [30]. The procedure included four types of target-source (i.e., CS-US) pairs: body images judged as similar or dissimilar were paired with positive and neutral pictures. However, depending on the contingency condition, the number of pairings differed. In the intervention condition, similar-positive and dissimilar-neutral pairs were in the majority, such that self-similar targets predicted the occurrence of positive images (i.e., a *similar body → positive* contingency was present). In the control condition, all types of pairs occurred equally often such that there was no *similar body → positive* contingency. Because we asked participants to categorize each positive or neutral source upon its occurrence, the successful encoding of the *similar body → positive* contingency could be inferred from systematic differences in response times and accuracy between predicted and unpredicted positive stimulus occurrences. Although different from previous implementations, this procedure had the advantage of using standardized stimuli throughout, including the body images, to avoid any unwanted visual distinctions or distractions that could interfere with encoding the intended *similar body → positive* contingency.

We hypothesized that a) the *similar body → positive* contingency would predict shorter response times and higher accuracy regarding positive image categorization in the intervention compared to the control condition and that b) these differences, as an index of *similar body → positive* contingency learning, would predict improvements in current body dissatisfaction due to the pairing procedure (i.e., we hypothesized that the effect of condition assignment on body dissatisfaction improvement is mediated by differences in contingency learning induced by the differences between the conditions). Moreover, because previous research showed that pre-existing, trait-like body image concerns could increase the impact of pairing procedures on state-like body dissatisfaction improvements [20–22], we further explored whether c) the effect of *similar body → positive* contingency learning on current body dissatisfaction improvements would increase with increasing levels of pre-existing body image concerns.

## Materials and methods

### Participants and design

We recruited 182 adult women (*M*_age_ = 31.4, age range: 18–64 years) for an online study from university and social network forums and among colleagues, friends, and acquaintances, to be randomly assigned to the conditions of a 2 (group: intervention vs. control) between-participants design. To homogenize and align the sample with previous studies [20, 24], we excluded women who disclosed a history of an eating disorder or who were currently receiving treatment for disordered eating. We initially envisioned including only healthy women with at least mild body image concerns, but dropped this criterion due to validity concerns (i.e., we intended to use a response toward a single question, which had not been validated) and because limiting the variability in the sample reduces the power for detecting moderations.

We targeted a minimum sample size of N = 128 to achieve a power of 1−β = 0.80 to detect a medium-sized difference (f = 0.25) between intervention vs. control condition for body dissatisfaction changes at α = 0.05. All data were collected from April 2022 to January 2023. Data and materials can be obtained from the corresponding author upon request. We report all measures, manipulations, exclusions and deviations from pre-registration (see Declarations).

### Measures and procedure

We implemented the study in jsPsych [33]. Upon accessing the study’s website, participants were randomly assigned to the intervention or control condition and read the study information and consent forms. Study participation required a physical keyboard. After providing informed consent, participants self-reported their age, weight, height, regular medications via open-ended questions and gender, language ability, years of education, dominant hand, pregnancy status, history of eating disorders, current eating disorder treatment, and previous study participation via multiple-choice questions. We then assessed, in random order, disordered eating symptoms via the German short version of the Eating Disorder Examination-Questionnaire (EDE-Q8, [34] as a control measure [20–23, 25, 26] and, more importantly, pre-existing trait-like body image concerns as a potential moderator of pairing procedure effects using the German Body Shape Questionnaire (*Fragebogen zum Figurbewusstsein*, BSQ; [35]. The BSQ´s 34 items assessed thoughts and concerns about one’s body shape during the past 28 days on a scale from 1 (*never*) to 6 (*always*), with the sum score serving as a validated measure of (trait-like) body image concerns.

### Target body selection

After completing the initial assessments, participants commenced to the main procedure. First, participants rated 44 standardized, black-and-white photographs of eleven women’s bodies (without heads and in neutral underwear), taken from four perspectives (standing-front, standing-back, standing-side, and sitting-side; for examples, see [36]. The models’ Body-Mass-Index (BMI) ranged from 13.8 to 61.3 kg/m^2^, and participants rated *How similar does this body looks to your body?* on a visual analog scale (VAS) ranging from -100 (not similar at all) to 100 (highly similar). We used each participant’s ratings to select the bodies with the highest and the median average similarity to serve as similar and dissimilar target stimuli, respectively. We chose the median rather than the least similar body for comparison, as we expected severely underweight or overweight bodies always to receive the lowest similarity ratings. Selecting the least similar body may thus have unintentionally confounded similarity with a specific weight dimension.

### Baseline state body dissatisfaction

Next, participants completed the German Body Image State Scales (BISS, [37, 38], which was also used in recent studies [23, 24, 26] and thus served as this study’s change-sensitive measure of state-like body dissatisfaction. The mean across its six items measured the current (“right now, in this very moment”) evaluation and affect about the physical appearance and attractiveness using different 9-point scales (from − 4, *very dissatisfied/very unattractive/very much worse than usual* to 4, *very satisfied/very attractive/very much better than usual*). We coded the scale such that higher scores indicate a more positive body evaluation.

### Pairing task and contingency learning assessment

**Fig. 1:**
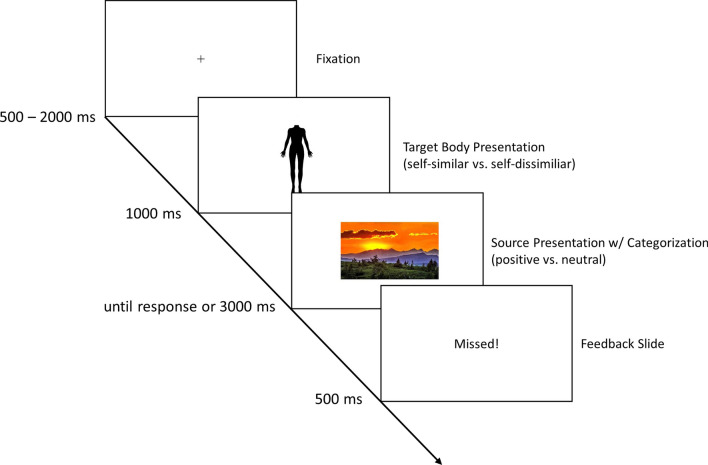
Trial sequence illustration of the pairing procedure. Each trial started with the display of a fixation, followed by a similar or dissimilar body image, then followed by the presentation of a positive or neutral target image. Participants were asked to categorize the target images upon presentation using their keyboard *c* and *m* keys. Trials were presented in random order

After completing the BISS, we explained that the next task would show photos of bodies as well as positive and neutral images. Each task trial (see Fig. 1) started with the display of a fixation cross presented for 500, 1000, 1500, or 2000 ms (selected at random). Next, one of four similar or dissimilar body images was displayed for 1000 ms (i.e., the two selected bodies across the four perspectives), followed by the presentation of a positive or neutral target image (e.g., of cute animals, happy people, landscapes vs. daily objects). We selected a total of 48 positive and 48 neutral target images from various validated databases [39–41]. Upon presentation of the positive or neutral image, participants were asked to categorize it as either positive or neutral using their keyboard’s *m* and *c* keys, respectively. This ensured focusing attention on the valence of the source stimuli, which has been known to strengthen pairing procedure effects [32]. The categorization further allowed us to measure contingency learning behaviorally [30]. If categorized incorrectly, or if no response was given within 3000 ms, an error message was displayed for 500 ms before advancing to the next trial (a blank slide was displayed for correct responses). In total, the task comprised 96 trials, presented in random order, such that each positive or neutral image was displayed only once. The task took approx. 8 min to complete.

Critically, in the intervention condition, pictures of the self-similar body were followed mainly by positive images (40 vs. 8). In contrast, pictures of the self-dissimilar body were followed less often by positive than neutral images (8 vs. 40). Thus, the *similar body → positive* contingency was ϕ = 0.67 in the intervention condition. In the control condition, there was no contingency between self-similarity and the valence of the source pictures (24 trials per each combination; *similar body → positive* contingency ϕ = 0). However, the overall number of similar and dissimilar body images did not differ between conditions, such that the total number of stimulus exposures and response base rates remained identical.

### State body dissatisfaction post-intervention and conclusion

After completing the pairing procedure, participants next completed the BISS for a second time to detect induced changes in body dissatisfaction. Finally, we asked participants to rate *How attractive do you find each body?* on a VAS ranging from − 100 (not at all attractive) to 100 (very attractive) for each picture presented for target body selection. We collected these ratings to explore any potential effects of the procedure on a perceived shift in body attractiveness [36]. After these ratings, participants were thanked and dismissed.

### Data aggregation and analysis

We extracted relative frequencies and median latencies of correct responses (i.e., response accuracy and response time in ms) for each participant from the data collected during contingency learning. These were submitted to two separate 2 (group: intervention vs. control) × 2 (target body: similar vs. dissimilar) × 2 (source valence: positive vs. neutral) analyses of variance (ANOVAs), with target body and source valence as repeated-measures factors, to assess the predicted effect of contingency learning.

The questionnaires showed high levels of internal consistency (Cronbach’s α = 0.96, 0.91, and 0.91/0.92 for the BSQ, EDE-Q8, and BISS baseline/post-intervention, respectively) and were thus aggregated according to their convention. We compared baseline scores (as well as participant characteristics) between conditions, and before and after the intervention with independent samples *t*-tests. Following recommendations [42], the effect on BISS scores post-intervention was examined in a 2 (group: intervention vs. control) analysis of covariance (ANCOVA), with baseline scores as covariate, as well as in a mediation analysis with contingency learning as a mediator and trait-like body image concerns as a moderator of the effect of contingency learning on current body dissatisfaction improvement (again, baseline BISS scores were included as covariate). We initially intended to include BSQ scores as a covariate in the ANCOVA, but omitted reporting this analysis as it yielded comparable effects, further moderations were described as exploratory analyses in the study registration.

The significance level for all analyses was set at *p* ≤ 0.05. Post hoc pairwise comparisons report Bonferroni-adjusted *p*-values for multiple comparisons. Effect sizes are reported as *η*_*p*_^*2*^. Variable values are reported as means and standard deviations (SDs). The data were aggregated and analyzed with IBM SPSS 28 [43]. We used PROCESS v4.2 model 14 [44] with boot-strapped (10,000 samples) bias-corrected 95% confidence interval (CI) for moderated mediation analysis. The effects of the moderator were evaluated at its mean (M) and at M ± 1 SD.

## Results

### Participant characteristics

We analyzed the data from 173 women (*M*_age_ = 30.9, age range: 18–64 years) assigned to the intervention (n = 84) and control condition (n = 89), respectively, after excluding participants that did not fulfill the inclusion criteria. The included sample sociodemographic and baseline characteristics are summarized in Table 1. The conditions did not differ in age, BMI, BSQ scores, EDE-Q8 scores, or baseline BISS scores, all *p*s > 0.35.

**Table 1:** Participant sociodemographic and baseline descriptive statistics (mean and SD or n and percentage)

Parameter	Total	Intervention	Control
Age	31.0 (12.5)	30.1 (12.1)	31.9 (12.9)
BMI	23.7 (4.7)	23.5 (4.2)	24.0 (5.1)
Gender			
Female	173	84	89
Pregnancy	0	0	0
German language			
First language	168 (97.1%)	82 (97.6%)	86 (96.6%)
Fluent	5 (2.9%)	2 (2.4%)	3 (3.4%)
Education			
12 years or more	152 (87.9%)	72 (85.7%)	80 (89.9%)
Less than 12 years	21 (12.1%)	12 (14.3%)	9 (10.1%)
Dominant hand			
Right	156 (90.2%)	75 (89.3%)	81 (91.0%)
Left	16 (9.2%)	8 (9.5%)	8 (9.0%)
Both	1 (0.6%)	1 (1.2%)	0 (0.0%)
BSQ score	77.1 (29.6)	78.1 (29.5)	76.1 (29.9)
EDE-Q8	1.8 (1.4)	1.8 (1.4)	1.8(1.4)
BISS (baseline)	0.9 (1.5)	0.9 (1.5)	0.9 (1.6)
BISS (post-intervention)	1.0 (1.5)	1.0 (1.4)	1.00 (1.6)

*BMI* body mass index in kg/m^2^; *BSQ* body shape questionnaire (*Fragebogen zum Figurbewusstsein*); *EDE-Q8* eating disorder examination-questionnaire short version; *BISS* body image state scale

### Contingency learning

**Fig. 2:**
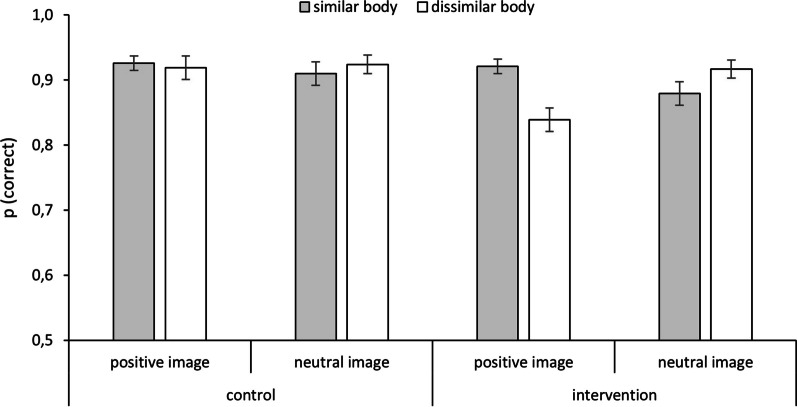
Relative frequencies of correct responses during *similar body → positive* contingency learning. Similar bodies predicted the occurrence of positive images in the intervention condition; there was no contingency between type of body and affective image in the control condition

Mean response accuracies are depicted in Fig. 2. The mixed-measures ANOVA revealed a group × target body interaction, *F*(1, 171) = 5.79, *p* = 0.02, *η*_*p*_^*2*^ = 0.03, and a target body × source valence interaction, *F*(1, 171) = 14.82, *p* < 0.001, *η*_*p*_^*2*^ = 0.08, which were qualified by the group × target body × source valence interaction, *F*(1, 171) = 7.23, *p* = 0.008, *η*_*p*_^*2*^ = 0.04; all other *F*s < 3.1, all *p*s > 0.08. As predicted, pairwise comparisons showed an increased frequency of correct responses for positive images in the intervention condition following similar bodies vs. dissimilar bodies, *p* < 0.001. Likewise, we found a decreased frequency of correct responses for neutral images following similar vs. dissimilar bodies, *p* = 0.014. In other words, response accuracies in the intervention condition reflected the manipulated *similar body → positive* contingency, with predicted source stimulus occurrences categorized more accurately than non-predicted occurrences. In comparison, response accuracy for positive and neutral images did not differ in the control condition as a function of the target body, *p*s > 0.33, consistent with the absence of a *similar body → positive* contingency in that condition.

A second mixed-measures ANOVA of response latencies revealed that positive images were correctly categorized faster than neutral images (*M* = 847 ms, *SD* = 216 vs. *M* = 884 ms, *SD* = 210), *F*(1, 170) = 22.57, *p* < 0.001, *η*_*p*_^*2*^ = 0.12. Other effects were not significant, all other *F*s < 2.5, *p*s > 0.11.

### Changes in state body dissatisfaction

BISS scores increased from baseline to after the intervention (*M*_*t1*_ = 0.89, *SD* = 1.54 vs. M_t2_ = 0.99, *SD* = 1.51), *t*(172) = 2.16, *p* = 0.03, *d* = 0.17, but the ANCOVA did not find an advantage of the intervention over the control condition post-intervention (*M* = 1.01, *SD* = 0.61 vs. *M* = 0.98, *SD* = 0.61, respectively), *F*(1, 170) = 0.10, *p* = 0.76. However, we hypothesized that pairing effects on state body dissatisfaction should be driven by successfully encoding a *similar body → positive* contingency (and we also intended to explore that pre-existing body image concerns further moderate this effect). We therefore investigated the hypothesized mediation pattern next. For this analysis, we calculated the individual differences between correct categorizations of positive images following similar vs. dissimilar target bodies as the index of *similar body → positive* contingency learning.

**Fig. 3:**
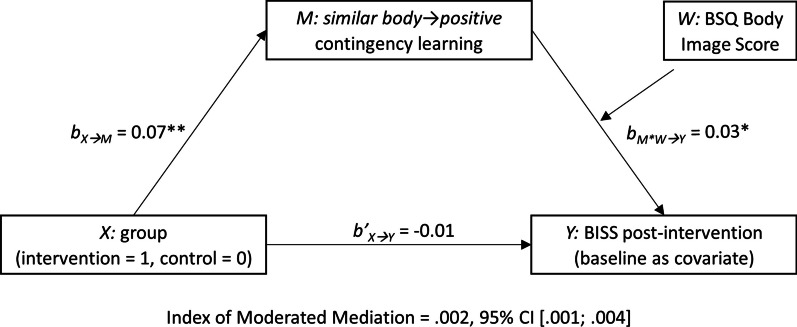
The moderated mediation model with group as predictor *X* (coded 1 0, for intervention and control conditions, respectively), *similar body → positive* contingency learning as mediator *M*, BISS post-intervention scores as criterion *Y* (with baseline scores as covariate), and BSQ body image scores as moderator *W* of the effect of contingency learning on BISS changes. The boot-strapped model confirmed that a positive effect of the intervention on contingency learning improved body dissatisfaction, but only among individuals with higher body image concerns

**Fig. 4:**
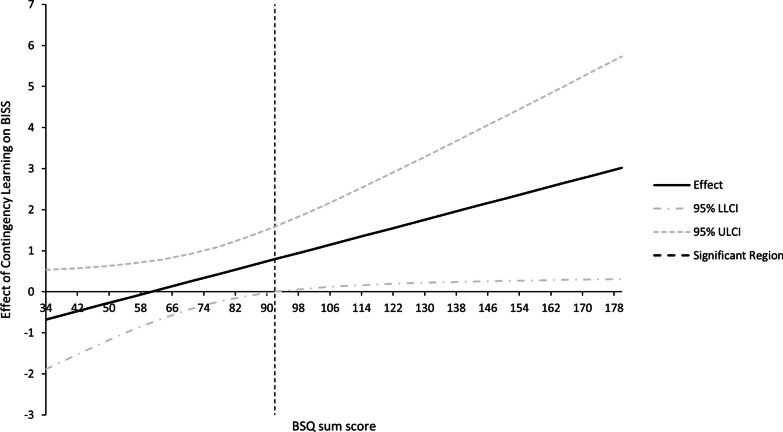
Johnson–Neyman Significance Regions for the Conditional Effects of Contingency Learning on State Body Dissatisfaction at Different Values of the Body Shape Questionnaire (BSQ). The effect is significant at BSQ values above the dotted, vertical line. LL = lower level, UL = upper level, CI = confidence interval

The moderated mediation model with group as predictor *X* (coded 1 0, for intervention and control conditions, respectively), *similar body → positive* contingency learning as mediator *M*, BISS scores post-intervention as criterion *Y* (with BISS baseline as covariate), and BSQ scores as moderator *W* of the effect of contingency learning, is displayed in Fig. 3. Consistent with the previous analysis, group predicted *similar body → positive* contingency learning, *b*_*X*_*→*_*M*_ = 0.07, *p* < 0.001. Contingency learning, in turn, interacted with BSQ scores to predict BISS change, *b*_M*W_*→*_*Y*_ = 0.03, *p* = 0.04, *R*^2^_*Change*_ = 0.004. Simple slopes showed that, at lower and mean BSQ levels, the effects of contingency learning on BISS change were not significant, *b*_*M*_*→*_*Y*_ = − 0.34, *p* = 0.49 and *b*_*M*_*→*_*Y*_ = 0.42, *p* = 0.22, respectively. However, at higher BSQ levels, an increase in contingency learning predicted improvements in BISS scores, *b*_*M*_*→*_*Y*_ = 1.17, *p* = 0.03 (for Johnson-Neyman significance regions, see Fig. 4). The boot-strapped model confirmed the hypothesized pattern: The index of moderated mediation (IMM) was significant, *IMM* = 0.002, 95% CI [0.001; 0.004], with an indirect effect (IE) observed for the high BSQ level, *IE* = 0.09, 95% CI [0.01; 0.20], but not at mean and lower BSQ levels, *IEs* = 0.03, 95% CI [− 0.01; 0.08] and − 0.03, 95% CI [− 0.08; 0.02], respectively. There was no direct effect (DE) of group on BISS changes when controlling for the influence of the mediator and moderator, *DE* = − 0.01, 95% [− 0.20; 0.18].

### Further exploratory analyses

**Fig. 5:**
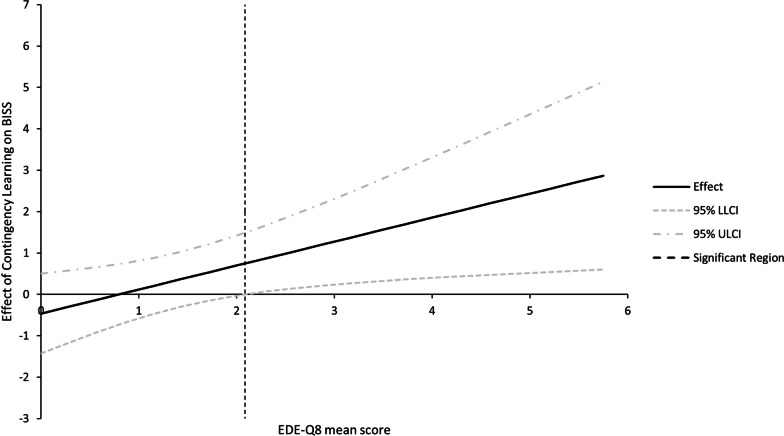
Johnson–Neyman Significance Regions for the Conditional Effects of Contingency Learning on State Body Dissatisfaction at Different Values of the Eating Disorder Examination-Questionnaire short version (EDE-Q8). The effect is significant at EDE-Q8 values above the dotted, vertical line. LL = lower level, UL = upper level, CI = confidence interval

Eating disorder psychopathology, as assessed by the EDE-Q8, correlated significantly with BSQ sum scores, *r*(171) = 0.80, *p* < 0.001, and BISS post-intervention, *r*(171) = -0.68, *p* < 0.001. Because several previous studies used EDE-Q scores for moderator analyses [20–22], we similarly explored moderated mediation patterns based on the EDE-Q8. When using the mean EDE-Q8 instead of the BSQ scores as moderator, an inferentially identical moderated mediation pattern emerged (see Table 2 and Fig. 5), suggesting an increased utility of pairing procedures with increasing levels of disordered eating symptoms. Other variables, such as participant age or BMI, showed no direct or indirect associations with the pairing procedure’s effects on changes in BISS. BSQ and EDE-Q8 scores showed no direct association with contingency learning across and within conditions, all *p*s > 0.10.

**Table 2:** Coefficient estimates with boot-strapped (10,000 samples) bias-corrected 95% confidence interval (CI) for the moderated mediation model based on disordered eating psychopathology

Path	b	SE	95% CI
X → M	0.07	0.02	[0.03; 0.12]
M × W → Y	0.58	0.25	[0.08; 1.08]
− SD	− 0.24	0.43	[− 1.08; 0.59]
M	0.59	0.35	[− 0.11; 1.28]
+ SD	1.41	0.5	[0.28; 2.54]
*Direct and indirect effects*			
DE	0.00	0.09	[− 0.19; 0.19]
IMM	0.04	0.03	[0.01; 0.11]
IE at − SD	− 0.02	0.02	[− 0.07; 0.02]
IE at M	0.04	0.03	[0.00; 0.12]
IE at + SD	0.11	0.06	[0.01; 0.26]

X = predictor variable group (coded 1 0, for intervention and control conditions); M = mediator variable *similar body → positive* contingency learning; Y = criterion Body Image State Scale scores post-intervention (with baseline as covariate); W = moderator variable Eating Disorder Examination-Questionnaire short version mean score; DE = direct effect; IMM = Index of Moderated Mediation; IE = indirect effect at different levels of the moderator; M = mean; SD = standard deviation. Model estimates are based on PROCESS v4.2 model 14 [44] with boot-strapped (10,000 samples) bias-corrected 95% confidence interval (CI)

Finally, we investigated the potential effects on participants’ attractiveness ratings. A 2 (group) × 2 (target body) mixed-measures ANOVA showed that bodies selected as similar were rated as more attractive than bodies at median similarity (*M* = 129, *SD* = 44.20 vs. *M* = 80.68, *SD* = 55.34), *F*(1, 171) = 66.08, *p* < 0.001,* η*_*p*_^*2*^ = 0.28. Attractiveness ratings were not directly affected by the group condition, all *F*s < 1, all *p*s > 0.42. However, additional exploratory moderated-mediation analyses showed that the difference in attractiveness between self-similar and self-dissimilar bodies increased as a function of group with increasing contingency learning, for individuals with high BSQ or EDE-Q8 scores (see Additional file 1). In other words, we observed a moderated-mediation pattern for the direct evaluation of body images parallel to changes in state body dissatisfaction. This suggests that a more positive evaluation of self-similar bodies could indeed be responsible for the observed improvements in body dissatisfaction.

## Discussion

The present study investigated the effect of a newly designed pairing procedure to improve current body dissatisfaction in women. Based on the idea that pairing effects could be partially driven by successfully encoding an *own body → positive* contingency, we predicted that learning a *similar body → positive* contingency would reduce current body dissatisfaction. Although we did not find that the *similar body → positive* contingency of pairings directly reduced body dissatisfaction levels, we found that participants reliably encoded the *similar body → positive* contingency in the intervention condition, and that body image improved for all participants (likely reflecting a mere body exposure effect; [45, 46]. Moreover, and as predicted, mediation analyses showed that learning the *similar body → positive* contingency, in turn, predicted current body dissatisfaction improvements for women, but only for those with high trait-like body image concerns or high disordered eating psychopathology.

The evidence for mediation reiterates the importance of assessing the mechanisms underlying the effects of pairing procedures. We proposed that learning (or failing to learn) the intended *target → source* contingency drives evaluative pairing effects, and speculated that similar previous studies (also different in several ways) may have failed to observe the effect of pairing participants' body images with other positive stimuli due to participants failing to learn the manipulated contingency [15]. Of course, we cannot verify these speculations a posteriori, and there are multiple mechanisms by which pairing procedures may exert a positive effect [19]. Still, the present findings support focusing efforts on improving or at least measuring relevant contingency learning in future applications.

Moreover, our findings are consistent with previous observations that the positive effects of pairing procedures on body dissatisfaction states are pronounced for individuals with pre-existing body image concern traits [20]. The moderated mediation patterns found here showed that only women with BSQ body image scores larger than 92, or EDE-Q8 scores larger 2.1, benefitted from successfully encoding the *similar body → positive* contingency (coincidentally, BSQ scores above 90 denote significant body image concerns according to normative data [47]. At the same time, many participants with low trait concerns (e.g., BSQ < 90) were still less than fully satisfied with their bodies prior to the intervention (*M* = 1.4 out of − 4 to + 4 at BISS baseline), implying that the moderation is not merely an artefact of regression to the mean. Thus, successful *body → positive* contingency learning may itself not be sufficient to incur improvements in state-like body dissatisfaction, and its potential for positive effects likely depends on further boundary conditions. It has been speculated that increased sensitivity to social feedback heightens body image concerns and, in turn, explains the increased efficacy of pairing procedures—as a way of mimicking social feedback—in improving body dissatisfaction [20]. Likewise, pre-existing body image concerns themselves could heighten attention towards comments, opinions, and reactions of others, that is, direct attention towards external information, and thus directly explain the increased effects of pairing procedures. The specific mechanism explaining the observed moderation thus still needs to be explored.

### Limitations and avenues for future research

Despite the data supporting an overall beneficial effect of pairing procedures, it is important to note several limitations. First, while we can assume that contingency-consistent differences in categorization performance reflect contingency learning, the absence of these effects does not necessitate the absence of learning. Besides the requirement of immediately responding to the stimuli, we did not include further attention checks and thus cannot rule out that aspects other than lower contingency learning contributed to the observed effects. Second, we recruited participants largely among German university students, who are likely White, female, more affluent, and more educated compared to the general population [48]. Although students are not exempt from experiencing body dissatisfaction, it is important to note that sociodemographic features such as gender and ethnicity can affect body image concerns and related health issues [49, 50]. As we did not explicitly assess participants’ ethnicities, we cannot conclude that the here observed effects will generalize towards more diverse samples without gathering further data. Relatedly, we must note that presenting bodies as “headless”, which was an unintended side-effect of using already existing stimulus materials, could have contributed to an “objectified” view of one’s own body, potentially further limiting the generalizability of our findings. Moreover, our study has thus far been limited to investigating short-term effects on body dissatisfaction improvements. Although pairing effects are relatively resistant toward extinction [51], thus far only a few studies demonstrated more long-term effects of pairing procedures for improving body image (up to 12 weeks after intervention [21, 22]. Finally, we only investigated the effect of a single session,the effect of repeated training sessions (i.e., the dosing level) needs to be further explored.

## Conclusions

Nonetheless, our findings point toward the relevancy of further exploring the utility of pairing procedures. *Similar body → positive* contingency learning improved current body dissatisfaction in individuals with normatively high body image concerns [47], which suggests these procedures could specifically benefit their intended audience. Moreover, the improvements were observed in addition to an overall effect of mere body exposure, which could suggest pairing procedures may ultimately augment other existing intervention strategies. Thus, further exploration of this line of research is warranted.

### Supplementary Information

**Additional file 1**. Indirect Effects of Pairings on Body Attractiveness Ratings.

## Data Availability

Data and materials can be obtained from the corresponding author upon request.

## Abbreviations

BISS: Body image states scale
BMI: Body mass index
BSQ: Body shape questionnaire
CS: Conditioned stimulus
DE: Direct effect
EDE-Q8: Eating disorder examination-questionnaire short version
IE: Indirect effect
IMM: Index of moderated mediation
US: Unconditioned stimulus
