# Supplementary material for: How evaluative pairings improve body dissatisfaction in adult women: evidence from a randomized-controlled online study
Source: J Eat Disord. 2024 Jan 24;12:18. doi: 10.1186/s40337-024-00975-4 (PMC10809437; doi:10.1186/s40337-024-00975-4)
Supplement: Supplementary file 1 — Additional file 1. Indirect Effects of Pairings on Body Attractiveness Ratings. [file 40337_2024_975_MOESM1_ESM.docx]

**Indirect Effects of Pairings on Body Attractiveness Ratings**

After completing the pairing procedure and post-intervention primary assessments (see the main manuscript), participants were asked to rate *How attractive do you find each body?* on a VAS ranging from -100 (not at all attractive) to 100 (very attractive) for each picture presented for target body selection. We collected these ratings to explore any potential effects of the procedure on a perceived shift in body attractiveness (Horndasch et al., 2015).

Attractiveness ratings were not directly affected by the group condition, but additional exploratory moderated-mediation analyses showed that the difference in attractiveness between self-similar and self-dissimilar bodies increased as a function of group with increasing contingency learning, for individuals with high BSQ or EDE-Q8 scores. Results of the moderated mediation models with group as predictor *X* (coded 1 0, for intervention and control conditions, respectively), *similar body→positive* contingency learning as mediator *M*, the difference in attractiveness between self-similar and self-dissimilar bodies as criterion *Y*, and either BSQ or EDE-Q8 scores as moderator *W* of the effect of contingency learning, are summarized in **Tables S1** and **S2**. In both analyses, contingency learning interacted with BSQ and EDE-Q8 scores to predict more favorable evaluation of the self-similar body over the self-dissimilar body, *b_M*W🡪Y_* = 3.41, *p* = .03, *R²_Change_* = .03 and *b_M*W🡪Y_* = 73.55, *p* = .01, *R²_Change_* = .03, respectively. Thus, we observed moderated-mediation patterns for the direct evaluation of body images analogues to changes in state body dissatisfaction, suggesting that a more positive evaluation of self-similar bodies could indeed be responsible for the observed body dissatisfaction improvement.

**Table S1**

*Coefficient Estimates with boot-strapped (10,000 samples) bias-corrected 95% confidence interval (CI) for the Moderated Mediation Model based on the Body Shape Questionnaire*

| **Path** | **b** | **SE** | **95% CI** |
| --- | --- | --- | --- |
| X→M | 0.07 | 0.02 | [0.03; 0.12] |
| M×W→Y | 3.4 | 1.51 | [0.43; 6.38] |
| - SD | 28.50 | 57.20 | [-84.42; 141.42] |
| M | 129.62 | 40.60 | [49.46; 209.78] |
| +SD | 230.75 | 63.39 | [105.59; 355.90] |
| *Direct and Indirect Effects* | | | |
| DE | -13.36 | 11.55 | [-36.17; 9.44] |
| IMM | 0.25 | 0.15 | [-0.00; 0.56] |
| IE at - SD | 2.12 | 4.05 | [-4.43; 11.94] |
| IE at M | 9.62 | 3.85 | [3.32; 18.30] |
| IE at +SD | 17.12 | 7.17 | [5.06; 33.27] |

*Note.* X = predictor variable group (coded 1 0, for intervention and control conditions); M = mediator variable *similar body→positive* contingency learning; Y = difference in attractiveness between self-similar and self-dissimilar bodies (range: 0 – 200); W = moderator variable Body Shape Questionnaire sum score; DE = direct effect; IMM = Index of Moderated Mediation; IE = indirect effect at different levels of the moderator; M = mean; SD = standard deviation. Model estimates are based on PROCESS v4.2 model 14 (Hayes, 2022) with boot-strapped (10,000 samples) bias-corrected 95% confidence interval (CI).

**Table S2**

*Coefficient Estimates with boot-strapped (10,000 samples) bias-corrected 95% confidence interval (CI) for the Moderated Mediation Model based on Disordered Eating Psychopathology*

| **Path** | **b** | **SE** | **95% CI** |
| --- | --- | --- | --- |
| X→M | 0.07 | 0.02 | [0.03; 0.12] |
| M×W→Y | 73.55 | 29.52 | [15.27; 131.84] |
| - SD | 24.50 | 50.18 | [-74.57; 123.56] |
| M | 129.73 | 41.42 | [47.96; 211.51] |
| +SD | 234.97 | 66.95 | [102.80; 367.13] |
| *Direct and Indirect Effects* | | | |
| DE | -11.41 | 11.25 | [-33.63; 10.80] |
| IMM | 5.46 | 3.10 | [0.65; 12.69] |
| IE at - SD | 1.82 | 3.53 | [-4.52; 9.67] |
| IE at M | 9.63 | 4.06 | [3.05; 18.77] |
| IE at +SD | 17.44 | 7.73 | [5.38; 35.55] |

*Note.* X = predictor variable group (coded 1 0, for intervention and control conditions); M = mediator variable *similar body→positive* contingency learning; Y = difference in attractiveness between self-similar and self-dissimilar bodies (range: 0 – 200); W = moderator variable Eating Disorder Examination-Questionnaire short version mean score; DE = direct effect; IMM = Index of Moderated Mediation; IE = indirect effect at different levels of the moderator; M = mean; SD = standard deviation. Model estimates are based on PROCESS v4.2 model 14 (Hayes, 2022) with boot-strapped (10,000 samples) bias-corrected 95% confidence interval (CI).
